# Value of ^18^F-FDG PET/CT-based radiomics model to distinguish the growth patterns of early invasive lung adenocarcinoma manifesting as ground-glass opacity nodules

**DOI:** 10.1186/s13550-020-00668-4

**Published:** 2020-07-13

**Authors:** Xiaonan Shao, Rong Niu, Xiaoliang Shao, Zhenxing Jiang, Yuetao Wang

**Affiliations:** 1 Department of Nuclear Medicine, The Third Affiliated Hospital of Soochow University, Changzhou, 213003 China; 2 Changzhou Key Laboratory of Molecular Imaging, Changzhou, 213003 China; 3 Department of Radiology, The Third Affiliated Hospital of Soochow University, Changzhou, 213003 China

**Keywords:** Radiomics, Ground-glass opacity nodules, Histopathologic subtype, Invasive adenocarcinoma, ^18^F-FDG, PET/CT

## Abstract

**Background:**

To establish and validate ^18^F-fluorodeoxyglucose (^18^F-FDG) PET/CT-based radiomics model and use it to predict the intermediate-high risk growth patterns in early invasive adenocarcinoma (IAC).

**Methods:**

Ninety-three ground-glass nodules (GGNs) from 91 patients with stage I who underwent a preoperative ^18^F-FDG PET/CT scan and histopathological examination were included in this study. The LIFEx software was used to extract 52 PET and 49 CT radiomic features. The least absolute shrinkage and selection operator (LASSO) algorithm was used to select radiomic features and develop radiomics signatures. We used the receiver operating characteristics curve (ROC) to compare the predictive performance of conventional CT parameters, radiomics signatures, and the combination of these two. Also, a nomogram based on conventional CT indicators and radiomics signature score (rad-score) was developed.

**Results:**

GGNs were divided into lepidic group (*n* = 18) and acinar-papillary group (*n* = 75). Four radiomic features (2 for PET and 2 for CT) were selected to calculate the rad-score, and the area under the curve (AUC) of rad-score was 0.790, which was not significantly different as the attenuation value of the ground-glass opacity component on CT (CT_GGO_) (0.675). When rad-score was combined with edge (joint model), the AUC increased to 0.804 (95% CI [0.699–0.895]), but which was not significantly higher than CT_GGO_ (*P* = 0.109). Furthermore, the decision curve of joint model showed higher clinical value than rad-score and CT_GGO_, especially under the purpose of screening for intermediate-high risk growth patterns.

**Conclusion:**

PET/CT-based radiomics model shows good performance in predicting intermediate-high risk growth patterns in early IAC. This model provides a useful method for risk stratification, clinical management, and personalized treatment.

## Background

Currently, lung cancer is the leading cause of cancer-related deaths, accounting for 23% of all cancer deaths [1], and 80–85% of them are non-small cell lung cancer (NSCLC). With the broad application of thin-layer CT scanning technology and the continuous development of lung cancer screening programs, the detection rate of early lung adenocarcinoma with ground-glass nodules (GGNs) continues to increase [2]. In many aspects, primary lung adenocarcinoma is considered as a very heterogeneous tumor with different histopathology and disease processes [3]. According to the 2011 classification of adenocarcinoma proposed by the International Association for the Study of Lung Cancer, the American Thoracic Society, and the European Respiratory Society (IASLC/ATS/ERS) [4], the most common patterns should be identified as the predominant growth patterns of invasive adenocarcinoma (IAC), including five subtypes: lepidic, acinar, papillary, micropapillary, and solid. The use of predominant growth patterns not only helps to classify IAC into subtypes but also serves as a prognostic indicator independent of clinical stage [5, 6]. Among the first three most common growth patterns, the prognosis of acinar or papillary types is worse than lepidic [6, 7]. The confirmation of the IAC growth pattern before surgery is essential for the risk stratification of GGN and personalized treatment.

PET/CT has become the primary imaging method for lung cancer evaluation. It can be used to detect and locate the primary tumor, determine the disease stage, or evaluate the treatment effect [8, 9]. However, whether the preoperative ^18^F-fluorodeoxyglucose (^18^F-FDG) PET/CT can be used to predict the growth pattern of IAC is still unclear [7, 10, 11]. The maximum standardized uptake value (SUV_max_) depends on two factors, the level of glucose uptake, and the spatial distribution of tumor cells. These factors are determined by the growth pattern of each tumor type, which is affected by the proliferation potential of tumor cells. In 2015, Nakamura et al. [7] first clarified the relationship between SUV_max_ and individual adenocarcinoma subtypes. The average SUV_max_ of acinar or papillary types was higher than that of the lepidic type. Son et al. [10] found that although solid and acinar types showed higher SUV_max_ since most IACs were lepidic or acinar, there was no significant difference in SUV_max_ between the main types. Our previous study [11] also showed similar results as Nakamura et al. Although SUV_max_ is the only independent factor that can distinguish the growth patterns of IAC, its identification efficacy is still not ideal (AUC = 0.628).

Radiomics is an emerging field in which a large number of objective and quantitative imaging features are explored in order to select the features that are most relevant to clinical, pathological, molecular, and genetic features. This method can increase the accuracy of diagnosis and prognosis and improve treatment efficacy [12]. The potential of this approach is to quantify the characteristics of tissues or organs beyond the visual interpretation or simple metrics. The texture analysis performed on ^18^F-FDG PET/CT images has shown great value in diagnosing NSCLC [13, 14]. In this study, we extracted the texture features of PET and CT images from the respective volume of interest (VOI) and established the PET/CT-based radiomics models to predict intermediate-high risk growth patterns of early IAC.

## Methods

### Patient selection

**Fig. 1:**
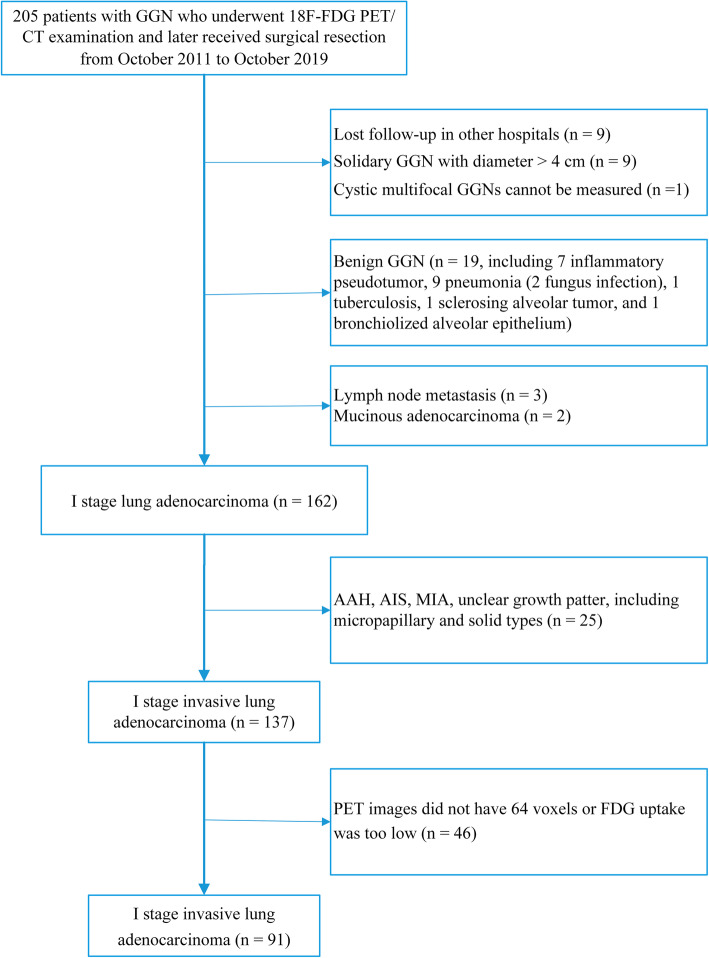
Flowchart of patient selection. GGN, ground-glass nodule, AAH atypical adenomatous hyperplasia, AIS adenocarcinoma in situ, MIA minimally invasive adenocarcinoma

In this retrospective single-center study, we enrolled 205 patients with GGN who underwent ^18^F-FDG PET/CT in our department and later received surgical resection from October 2011 to October 2019. The classification of surgical pathology is based on the 2011 classification of lung adenocarcinoma published by IASLC/ATS/ERS [4]. This study was approved by the institutional ethics committee for retrospective analysis and did not require informed consent. Inclusion criteria: (1) stage I lung adenocarcinoma; (2) lung nodules manifested as GGN; (3) lesion size ≤ 4 cm; (4) PET/CT examination before surgery; (5) radical resection on tumor; and (6) PET/CT and surgery were completed within 1 month. Exclusion criteria: (1) diameter of GGN > 4 cm; (2) lesion with poor image quality or low FDG uptake that were difficult to measure; (3) patients who had received anti-tumor treatment; (4) lung adenocarcinoma stage > I; (5) history of severe liver disease, diabetes, or cancer; (6) postoperative pathological subtypes of atypical adenomatous hyperplasia, adenocarcinoma in situ, or minimally invasive adenocarcinoma; (7) unclear growth patterns or rare growth patterns (such as micropapillary and solid types); and (8) PET images did not have enough voxels (64 voxels) required by the software or metabolic volume after segmentation lower than 2.5 ml. The patient selection process was shown in Fig. 1.

### FDG PET/CT image acquisition

Image acquisition protocols were described according to the Imaging Biomarker Standardization Initiative (IBSI) Reporting Guide [15]. All the program details were described in the electronic supplementary material 1. Within 1 month before surgery, the patients received an ^18^F-FDG PET/CT examination (Biograph mCT 64, Siemens, Erlangen, Germany). Based on the European Association of Nuclear Medicine (EANM) guideline 1.0 (version 2.0 was released in February 2015) [16], the ^18^F-FDG PET/CT images were acquired at 60 ± 5 min after ^18^F-FDG injection. All PET/CT images were reconstructed on a processing workstation (TureD software, Siemens Healthcare). CT data were used to perform attenuation correction on PET image, and the corrected PET image was fused with the CT image.

### Radiomic feature extraction

The LIFEx software (version 5.10, http://www.lifexsoft.org) was used to extract the texture features of PET/CT images from the VOI of the lesions [17]. The patients’ PET/CT images in DICOM format were imported into the software. For PET images, experienced diagnostic physicians used the 40% and 70% threshold of SUV_max_ to semi-automatically set the target area of the lesion [18]. The VOI on the CT images was manually delineated and segmented slice-by-slice. The VOI covered the whole lesion, and large vessels and bronchus were excluded from the volume of the nodule. Considering the effect of different quantization levels on PET texture features, we set different higher bound of SUV (10 vs. 20) in the absolute resampling method. Finally, the software program automatically calculated and extracted 52 PET radiographic features and 49 CT radiographic features, which were provided in the supplementary material 1.

### The selection of radiomic feature and the establishment of the model

In this study, the number of radiomic features was large, but the number of cases was relatively small. To avoid model overfitting, we first used the Mann-Whitney *U* test to preselect the features with significant differences between acinar-papillary group and lepidic group (*p* value relaxed to < 0.10). Then, the least absolute shrinkage and selection operator (LASSO) algorithm was used to select the best features among the preselected features [19]. The LASSO algorithm added an L1 regularization term to the least-squares algorithm to avoid overfitting. It shrinks some coefficients and reduces others to exactly 0 via the absolute constraint. A model was generated using a linear combination of selected features that were weighted by their respective LASSO coefficients; the model was then used to calculate a radiomics signature score (rad-score) for each GGN based on the selected discriminating radiomic features. The receiver operating characteristic (ROC) curve and the area under the curve (AUC) were used to evaluate model performance.

### Statistical analysis

Continuous variables were expressed as mean ± standard deviation (SD) or median (25th to 75th percentiles), and categorical variables were expressed as frequency (%). Independent *t* tests or Mann-Whitney *U* tests were used to compare continuous variables, and the Pearson chi-square test and Fisher’s exact test were used to comparing categorical variables. Multi-factor logistic regression was used to establish the prediction model, and the most optimal model parameters were selected using the minimum Akaike’s information criterion (AIC). The Bootstrap resampling method (times = 500) recommended by the TRIPOD Reporting Specification [20] was used to internally validate the model and calculate the 95% confidence interval (CI) of the AUC. A correlation heat map between each selected feature was established using the Spearman rank correlation method. The nomogram of the model was drawn in order to visualize the prediction results of each patient. A calibration curve was also drawn to show the prediction accuracy of the nomogram. ROC curve was made for each model, and the AUC of different models were compared using the DeLong method [21]. The clinical effectiveness of the model was quantified and compared using the decision curve analysis (DCA) method, which evaluates the relative cost of false positives and false negatives based on threshold probabilities. By subtracting the proportion of false positives from the proportion of true positives, and weighing the relative cost of false positives and false negatives, we can get a net benefit. The following formula was used to calculate the net benefit of model-based decisions:

$$ \mathrm{Net}\;\mathrm{benefit}=\frac{\mathrm{True}\kern0.17em \mathrm{positives}}{\mathrm{n}}\hbox{-} \frac{\mathrm{Pt}}{1\hbox{-} \mathrm{Pt}}\times \frac{\mathrm{False}\kern0.17em \mathrm{positives}}{\mathrm{n}} $$

Where *n* is the total number of patients in the study, and Pt is the given threshold probability. All analyses were performed using R3.4.3 (http://www.R-project.org; software packages: glmnet, pROC, rms, dca. R). *P* < 0.05 was considered statistically significant. The patients with missing key parameters were excluded from the analysis, and their data were not estimated.

## Results

### Patient characteristics and general PET/CT parameters

Finally, this study included 91 patients with IAC (23 male and 68 female), with an average age of 61.8 ± 8.6 years, ranging from 38 to 80 years. Thirteen (14.3%) patients had a history of smoking. Among the 91 patients, 59 had solitary GGN, and 32 had multifocal GGN (total lesion number 173, median lesion number 3, ranging from 2 to 36). According to the IASLC/ATS/ERS adenocarcinoma classification and prognosis standard [6, 7], 93 GGNs were classified and divided into low-risk lepidic group (*n* = 18), and intermediate-high risk acinar-papillary group (*n* = 75, 65 acinar and 10 papillary).

The acinar-papillary group had significantly higher CT_GGO_ than the lepidic group (*P* = 0.014), and the lobulated edges were also more common in the acinar-papillary group (*P* = 0.022). The comparison of conventional PET/CT parameters between the two groups was shown in Table 1.

**Table 1:** Comparison of conventional PET/CT parameters between lepidic group and acinar-papillary group

Growth pattern group	Lepidic *n* = 18	Acinar-papillary *n* = 75	*P* value
Nodule type			
pGGN	4 (22.2%)	13 (17.3%)	0.630
mGGN	14 (77.8%)	62 (82.7%)	
Location			
Subpleural/perifissural	17 (94.4%)	74 (98.7%)	0.351
Parenchymal	1 (5.6%)	1 (1.3%)	
Shape			
Round/oval	10 (55.6%)	32 (42.7%)	0.324
Polygonal/irregular	8 (44.4%)	43 (57.3%)	
Edge			
Smooth	11 (61.1%)	24 (32.0%)	0.022
Lobular/spiculated	7 (38.9%)	51 (68.0%)	
Bronchial sign			
Natural	3 (16.7%)	8 (10.7%)	0.479
Dilated/distorted/cutoff	15 (83.3%)	67 (89.3%)	
Cystic appearance present	1 (5.6%)	13 (17.3%)	0.290
Pleural indentation present	14 (77.8%)	65 (86.7%)	0.344
Vascular convergence present	18 (100.0%)	73 (97.3%)	0.484
D_GGN_ (mm)	26.4 (21.0–28.9)	24.3 (19.2–28.4)	0.918
D_Solid_ (mm)	8.1 (3.7–15.2)	11.8 (5.6–16.1)	0.257
CTR	0.3 (0.2–0.6)	0.5 (0.3–0.7)	0.168
CT_GGO_ (HU)	− 545.5 (− 598.8–398.8)	− 425.0 (− 477.5–335.0)	0.014
SUV_max_	2.4 (1.7–3.3)	2.9 (2.1–4.3)	0.076

Except where otherwise indicated, data are number (%) of GGNs

*GGO* ground-glass opacity, *D*_*GGN*_ diameter of the GGN, *D*_*Solid*_ diameter of the solid component, *CT*_*GGO*_ attenuation value of the GGO component on CT, *SUV*_*max*_ maximum standardized uptake value

### Feature Extraction And Selection

The mean tumor volume segmented by the semi-automatically thresholding method (70 and 40% of SUV_max_) was 6.7 ± 2.3 ml (range 2.8–12.5 ml) and 7.2 ± 2.9 ml (range 3.0–17.0 ml). On the CT images manually delineated and segmented slice-by-slice, the mean tumor volume was 6.2 ± 4.7 ml (range 0.8–20.3 ml).

Under the higher bound of SUV 20, we compared the effects of 70 and 40% delineation thresholds (PET: 64 bins from 0 to 20) on PET preselected features. It was found that compared with the 40% threshold, although 70% threshold preselected more features (22 vs. 18), the PET score produced by the 40% threshold showed better discrimination (AUC = 0.735 vs. 0.707). Besides, we also found that SHAPE_Sphericity and GLZLM_ZLNU in PET features were robust to different thresholds (Supplementary Material 2).

At 40% delineation thresholds, we compared the effects of different higher bound of SUV (10 vs. 20) on PET preselected features. It was found that the preselected features did not change significantly (especially for conventional indices, first order features, and GLCM). The PET score produced by the higher bound of 20 showed better discrimination (AUC = 0.735), but the difference from the higher bound of 10 was very small (AUC = 0.712). Besides, we also found that SHAPE_Sphericity in PET features was robust to different higher bound of SUV (Supplementary Material 2). Therefore, the final PET parameters were 40% thresholds and 64 bins from 0 to 20, while CT used the default parameters.

**Fig. 2:**
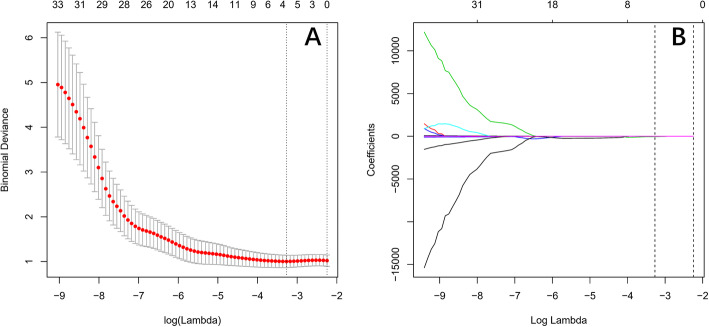
The best subset of radiomic features was extracted using the LASSO algorithm and 10-folds cross-validation. **a** The best feature was selected based on the AUC value. The black vertical line defines the best value of *λ*, and the model provides the best fit of the data. *λ* = 0.038 with log (*λ*) = − 3.2697 is selected as the optimal value. **b** The LASSO coefficient profiles of 38 radiomic features. The vertical line is the value selected by 10-fold cross-validation in **a**, where the best *λ* results in four nonzero coefficients of four selected features

LASSO algorithm and 10-folds cross-validation were used to extract the best subset of radiomic features, and four radiomic features were extracted (Fig. 2), which were SHAPE_Sphericity, GLZLM_ZLNU, HISTO_Kurtosis, and GLZLM_SZLGE. Among the above features, the first 2 are PET features, and the last 2 are CT features. The following formula was used to calculate the rad-score for each GGN:

$$ \mathrm{Rad}\hbox{-} \mathrm{score}=\hbox{-} 5.25465\times \mathrm{SHAPE}\_\mathrm{Sphericity}+0.0995\times \mathrm{GLZLM}\_\mathrm{ZLNU}\hbox{-} 0.28141\times \mathrm{HISTO}\_\mathrm{Kurtosis}\hbox{-} 40.16559\times \mathrm{GLZLM}\_\mathrm{SZLGE} $$

Table 2 shows the median and interquartile range of the four selected radiomic features and the calculated rad-score. There were significant differences in rad-score and the four selected features between the lepidic group and the acinar-papillary group (all *P* < 0.05).

**Table 2:** Comparison of four radiomic features and rad-score between lepidic group and acinar-papillary group

Growth pattern group	Lepidic *n* = 18	Acinar-papillary *n* = 75	*P* value
Rad-score	− 6.485 (− 6.698–6.142)	− 5.931 (− 6.283–5.466)	< 0.001
Features PET			
SHAPE_Sphericity	1.062 (1.040–1.099)	1.042 (0.998–1.069)	0.027
GLZLM_ZLNU	1.432 (1.050–2.377)	2.529 (1.333–5.773)	0.024
Features CT			
HISTO_Kurtosis	2.653 (2.352–3.945)	2.380 (2.151–2.754)	0.029
GLZLM_SZLGE	0.003 (0.002–0.004)	0.001 (0.001–0.003)	0.047

The value of each radiomic feature was expressed as median (25th to 75th percentiles)

*Rad-score* radiomics signature score

### Pairwise correlation between PET/CT radiomic features

**Fig. 3:**
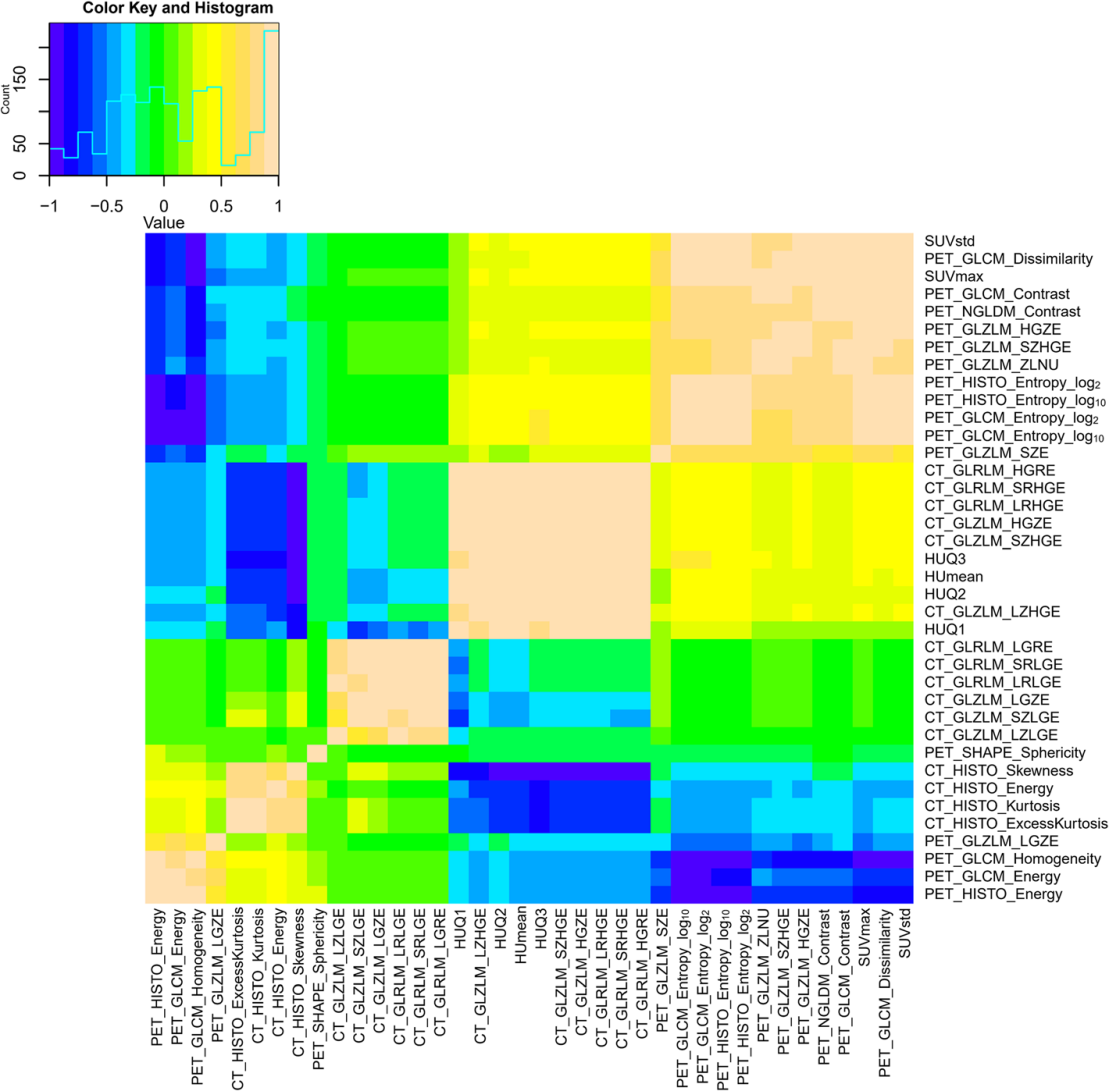
The Spearman rank correlation method was used to establish a correlation map between each preselected feature. This method integrates CT and PET images. The corresponding coefficients are shown in the heat map, where yellow and blue indicate positive and negative correlations, respectively

Figure 3 shows the correlation heat map of preselected PET/CT features, which illustrates the results of feature selection. The color keys and histogram bars in the upper left corner indicate the correlation between each image feature. A correlation equals to 0 indicates the best independence among the corresponding features, while a correlation equal to 1 or − 1 suggests a perfect correlation. We found that the four extracted PET/CT features were independent of each other (|r| all < 0.5), indicating that these features could convincingly represent the tumor features and the prediction model was reliable.

### Construction of joint model and personalized nomogram

We combined rad-score and conventional CT parameters (edge and CT_GGO_) to establish a multivariate logistic regression model (joint model) and used a non-parametric resampling method (Bootstrap resampling, times = 500) to perform internal verification.

The model is as follows:

$$ \mathrm{Logit}\ \left(\mathrm{P}\right)=13.90677+2.09540\times \mathrm{rad}\hbox{-} \mathrm{score}+0.86999\times \left(\mathrm{edge}=\mathrm{lobular}/\mathrm{spiculated}\right) $$

**Fig. 4:**
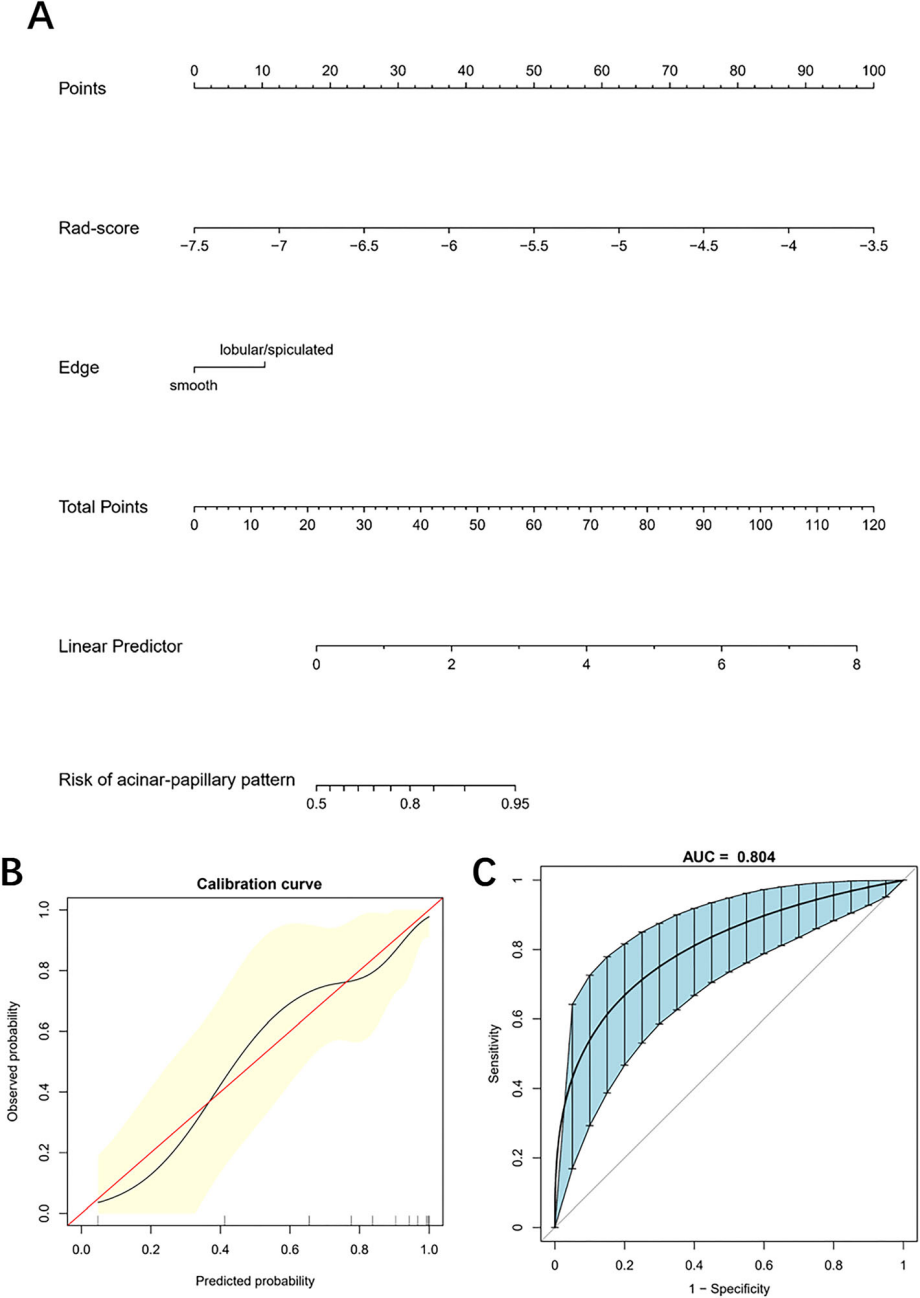
The nomogram and its performance. The nomogram, calibration curve, and ROC based on the joint model (rad-score and edge) were drawn (**a**–**c**). **b** The horizontal axis of the calibration curve is the predicted incidence of the acinar-papillary pattern, and the vertical axis is the observed incidence. The red diagonal line is the reference line, indicating that the predicted value equals to the observed value. The black line is the calibration curve, and the yellow areas on both sides represent 95% CI. **c** The ROC curve and 95% confidence interval of the joint model were drawn by the Bootstrap resampling (times = 500). Rad-score, radiomics signature score

The nomogram and a calibration curve of the joint model were drawn (Fig. 4a, b). There was good consistency between the predicted and observed values, and the ROC curve of the joint model showed an AUC of 0.804 (95% CI [0.699 – 0.895]) (Fig. 4c).

### Performance of radiomic features and conventional CT parameters

**Fig. 5:**
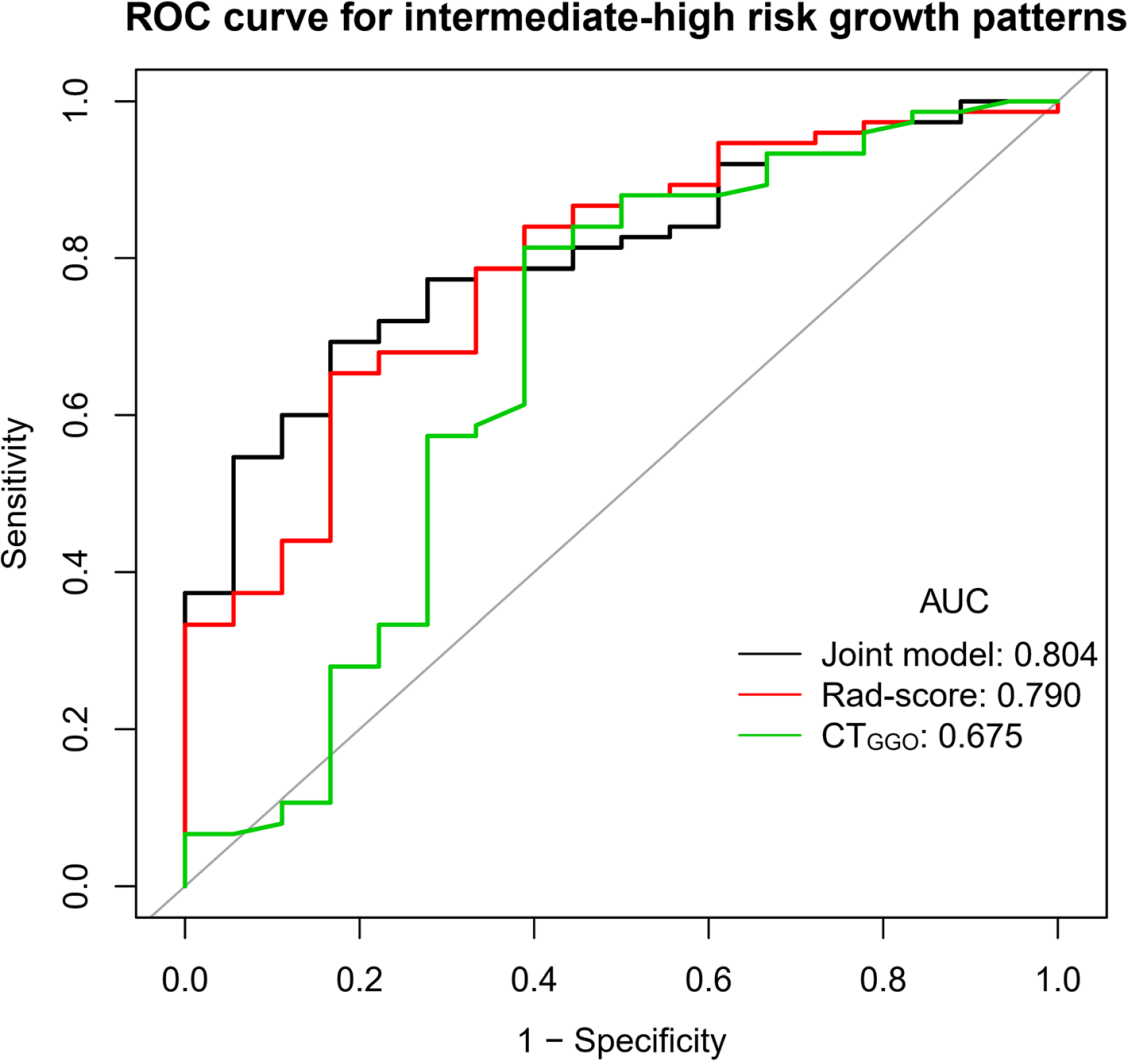
Comparison of the ROC curves of three models in predicting intermediate-high risk growth patterns of IAC. Rad-score, radiomics signature score; CT_GGO_, attenuation value of the GGO component on CT

To evaluate the performance of radiomic features in predicting GGN growth patterns, we compared the rad-score, CT_GGO_, and the joint model using ROC (Fig. 5). The prediction capabilities of the three models are listed in Table 3, including AUC, sensitivity, specificity, accuracy, positive likelihood ratio, and negative likelihood ratio. The results showed that the AUC of the joint model and rad-score were higher than CT_GGO_ (0.804 vs. 0.675 and 0.790 vs. 0.675), but the difference was not statistically significant (*P* = 0.109 and 0.132). There was also no significant difference between the joint model and the rad-score (*P* = 0.605).

**Table 3:** ROC analysis for three models

Test	AUC (95%CI)	Specificity	Sensitivity	Accuracy	PLR	NLR
CT_GGO_	0.675 (0.508–0.841)	0.611	0.813	0.774	2.091	0.306
Rad-score	0.790 (0.676–0.903)	0.833	0.653	0.688	3.920	0.416
Joint model	0.804 (0.699–0.895)	0.833	0.693	0.720	4.160	0.368

*Rad-score* radiomics signature score, *CT*_*GGO*_ attenuation value of the GGO component on CT, *PLR* positive likelihood ratio, *NLR* negative likelihood ratio

**Fig. 6:**
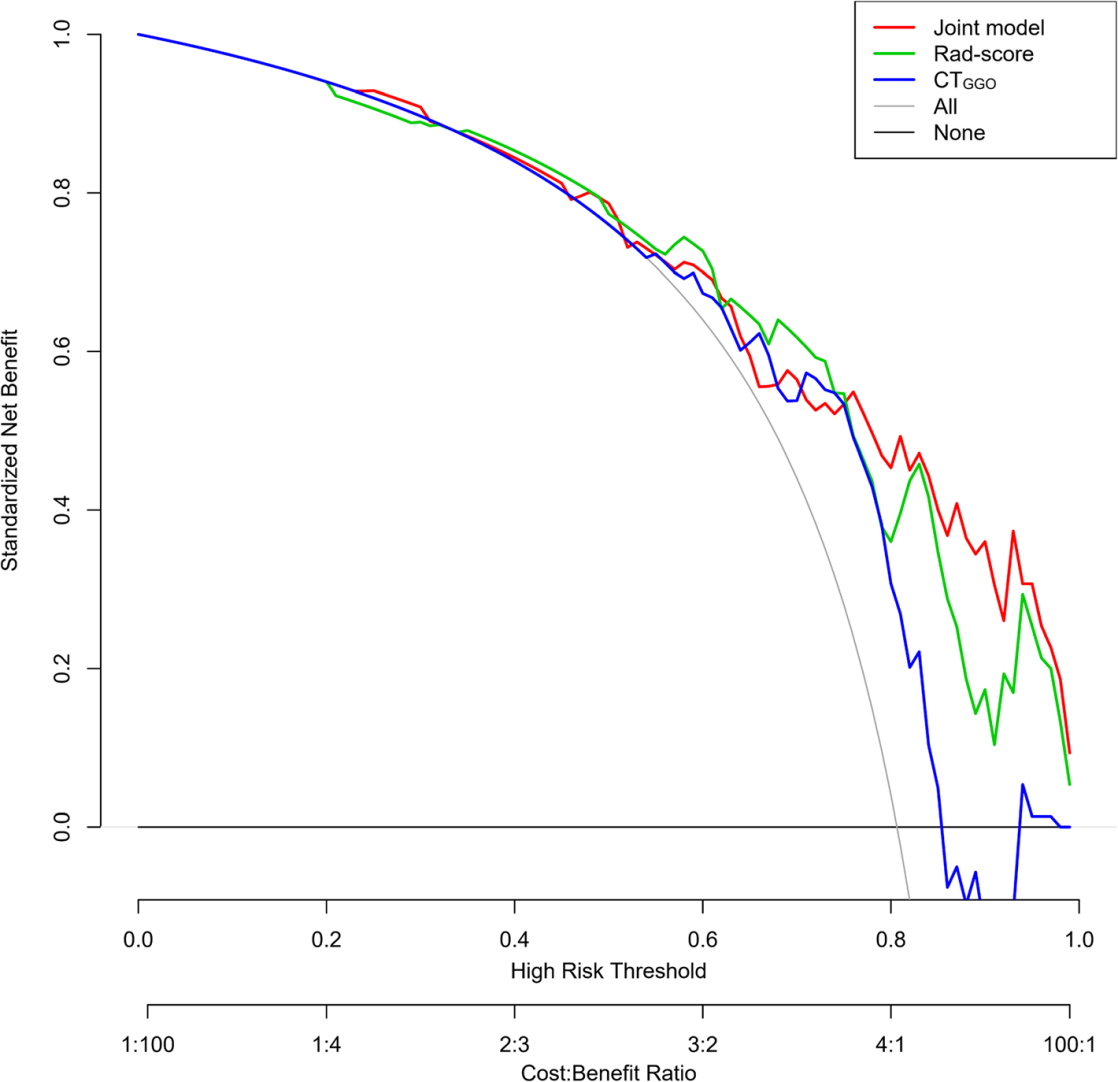
Decision curve analysis of three models in predicting the correct diagnosis of intermediate-high risk growth patterns of IAC. Rad-score, radiomics signature score; CT_GGO_, attenuation value of the GGO component on CT

Since the AUC of the joint model, rad-score, and CT_GGO_ were not significantly different, we introduced DCA in order to evaluate the performance of the three models (Fig. 6). Under the purpose of screening for intermediate-high risk growth patterns (sensitivity ≥ 0.800, threshold probability ranging from 0.73 to 0.98), the net benefit of the joint model was better than rad-score and CT_GGO_; similarly, under the purpose of confirming the diagnosis of intermediate-high risk growth patterns (specificity ≥ 0.833, threshold probability ranging from 0.30 to 0.59), there was no significant difference in net benefit between the three. Thus, the overall clinical value of the joint model was higher than the other two.

## Discussion

Given the established role of the growth pattern in the early lung adenocarcinoma with GGN, there is a need for non-invasive imaging methods. PET-based SUV_max_ is a commonly used parameter in the diagnosis of lung cancer. However, it ignores the relationships between two or more voxels, so diagnostic efficiency is not high. In this study, we built a model based on four preoperative radiomic features of ^18^F-FDG PET/CT images to predict the intermediate-high risk growth pattern in early IAC, and the model showed excellent predictive performance.

The four texture features, including two PET features and two CT features, are all related to image uniformity or heterogeneity. “Sphericity” is a tumor shape descriptor based on PET images, which quantifies the similarity of metabolic tumor volume (MTV) shape and spherical surface. It is entirely defined by the surface of the tumor and therefore only depends on the heterogeneity within the tumor. To a certain extent, segmentation depends on this heterogeneity. Apostolova et al. [22] studied “asphericity”, the antonym of “sphericity”, and found that asphericity is related to the growth, proliferation, and angiogenesis of NSCLC. Moreover, in adenocarcinoma (ADC), this correlation is much stronger than in squamous cell carcinoma (SCC). In predicting progression-free survival and overall survival, the prognostic power of asphericity is significantly higher than other PET-based parameters (SUV and MTV), clinical and molecular characteristics [22, 23]. Hyun et al. [24] used a machine learning algorithm with PET radiomic features to distinguish between ADC and SCC. They found that SCC’s GLZLM_ZLNU is significantly higher than ADC, indicating that SCC is more heterogeneous. Our results also found that sphericity was not easily affected by segmentation methods and quantization levels, which was consistent with the results of Oliver et al. [25], while GLZLM_ZLNU was also robust to different segmentation methods.

“Kurtosis” derived from the CT histogram reflects the gray distribution in the reaction area. In a practical application, Chae et al. [26] found that when analyzing GGN, higher kurtosis is a significant difference between preinvasive lesions and IAC. This is consistent with our result that kurtosis of the lepidic group was higher because preinvasive lesions are mainly based on lepidic growth. Besides, Tsubakimoto et al. [27] found that even in distinguishing ADC and SCC, kurtosis is not as strong as SUV_max_, but the diagnostic ability of kurtosis is still strong enough. In the heat map, we found that GLZLM_SZLGE had an excellent negative correlation with HU in conventional indices (especially HUQ1, which represents a low attenuation region; the correlation coefficient was close to − 1). Therefore, it can be considered that CT radiomic features contain the CT_GGO_ information, so in the end, CT_GGO_ did not enter the joint model. On HRCT, the GGO component of GGN can indicate a lepidic growth pattern [28]. The high CT attenuation values of pGGNs suggest IAC [29], and CT_GGO_ is an independent predictor of IAC [30, 31].

We found that the CT signs of the two groups with different IAC growth patterns were mostly overlapped. Among them, the edge was the most promising qualitative CT parameter, and the acinar-papillary group showed a higher proportion of lobulated edge than lepidic group. Lobulation is one of the characteristics of malignant GGN [32], and it can be used to predict the invasion of GGN [33]. Moreover, the rad-score that we developed showed a better ability to distinguish the growth patterns. When rad-score was combined with the edge, its clinical value was improved. Besides, we developed a nomogram based on rad-score and edge, which can visualize the prediction results and provide an easy-to-use method for personalized prediction of intermediate-high risk growth patterns.

Our study has some limitations: (1) Although we did internal validation, the single-center design and relatively small sample size may still impair the applicability of the model, especially when it does not include the highest-risk types: solid and micropapillary. Therefore, it is necessary to conduct a standardized multi-center study, expand the sample size, and conduct external validation. (2) This study did not consider the mutation status of EGFR, but the sub-solid nodules have a high EGFR mutation rate [34]. The subsequent studies should consider EGFR status as a confounding factor. (3) This study has preliminarily demonstrated the potential of radiomics models. In the future, machine learning or deep learning models should be established, in order to improve the predictive performance. (4) The heterogeneity of lung cancer has been shown to play an essential role in disease prognosis [35]. Due to the short follow-up time, the prognostic value of PET/CT radiomics models for different IAC growth patterns is unclear.

## Conclusions

In conclusion, the radiomics model based on preoperative ^18^F-FDG PET/CT has excellent prediction performance. This model provides a relatively accurate, convenient, and non-invasive method to predict the intermediate-high risk growth pattern of IAC, which is very useful in clinical practice and can be used for risk stratification and personalized treatment.

## Supplementary information

Additional file 1: Supplementary material 1. Report on image processing and radiomic features extraction

Additional file 2: Supplementary material 2. Significance of PET/CT texture features between lepidic group and acinar-papillary group

## Data Availability

The data supporting our findings are available upon request.

## Abbreviations

IAC: Invasive adenocarcinoma
GGNs: Ground-glass nodules
LASSO: Least absolute shrinkage and selection operator
ROC: Receiver operating characteristics
AUC: Area under the curve
NSCLC: Non-small cell lung cancer
IAC: Invasive adenocarcinoma
EANM: European Association of Nuclear Medicine
AIC: Akaike’s information criterion
CI: Confidence interval
DCA: Decision curve analysis
MTV: Metabolic tumor volume
ADC: Adenocarcinoma
SCC: Squamous cell carcinoma
EGFR: Epidermal growth factor receptor

## References

[1] Siegel RL, Miller KD. Cancer statistics, 2020. 2020;70:7-30. doi:10.3322/caac.21590. 10.3322/caac.21590 31912902

[2] Wiener RS, Gould MK, Arenberg DA, Au DH, Fennig K, Lamb CR (2015). An official American Thoracic Society/American College of Chest Physicians policy statement: implementation of low-dose computed tomography lung cancer screening programs in clinical practice. Am J Respir Crit Care Med.

[3] Gridelli C, Rossi A, Carbone DP, Guarize J, Karachaliou N, Mok T (2015). Non-small-cell lung cancer. Nat Rev Dis Prim.

[4] Travis WD, Brambilla E, Noguchi M, Nicholson AG, Geisinger K, Yatabe Y (2013). Diagnosis of lung adenocarcinoma in resected specimens: implications of the 2011 International Association for the Study of Lung Cancer/American Thoracic Society/European Respiratory Society classification. Arch Pathol Lab Med.

[5] Russell PA, Wainer Z, Wright GM, Daniels M, Conron M, Williams RA (2011). Does lung adenocarcinoma subtype predict patient survival? A clinicopathologic study based on the new International Association for the Study of Lung Cancer/American Thoracic Society/European Respiratory Society international multidisciplinary lung adenocarcinoma classification. J Thoracic Oncol.

[6] Warth A, Muley T, Meister M, Stenzinger A, Thomas M, Schirmacher P (2012). The novel histologic International Association for the Study of Lung Cancer/American Thoracic Society/European Respiratory Society classification system of lung adenocarcinoma is a stage-independent predictor of survival. J Clin Oncol.

[7] Nakamura H, Saji H, Shinmyo T, Tagaya R, Kurimoto N, Koizumi H (2015). Lung cancer (Amsterdam, Netherlands).

[8] Luketich JD, Friedman DM, Meltzer CC, Belani CP, Townsend DW, Christie NA (2001). The role of positron emission tomography in evaluating mediastinal lymph node metastases in non-small-cell lung cancer. Clin Lung Cancer.

[9] Ettinger DS, Wood DE, Aisner DL, Akerley W, Bauman J, Chirieac LR (2017). Non-small cell lung cancer, version 5.2017, NCCN clinical practice guidelines in oncology. JNCCN.

[10] Son BY, Cho S (2018). The maximum standardized uptake value of preoperative positron emission tomography/computed tomography in lung adenocarcinoma with a ground-glass opacity component of less than 30 mm. J Surg Oncol.

[11] Shao X, Niu R, Jiang Z, Shao X, Wang Y (2020). Role of PET/CT in management of early lung adenocarcinoma. AJR Am J Roentgenol.

[12] Gillies RJ, Kinahan PE, Hricak H (2016). Radiomics: images are more than pictures, they are data. Radiology..

[13] Zhang J, Zhao X (2019). Value of pre-therapy (18)F-FDG PET/CT radiomics in predicting EGFR mutation status in patients with non-small cell lung cancer.

[14] Jiang M, Zhang Y, Xu J, Ji M, Guo Y, Guo Y (2019). Assessing EGFR gene mutation status in non-small cell lung cancer with imaging features from PET/CT. Nucl Med Commun.

[15] Zwanenburg A, Leger S, Vallières M, Löck S. Image biomarker standardisation initiative. arXiv preprint arXiv:161207003. 2016.

[16] Boellaard R, Delgado-Bolton R, Oyen WJ, Giammarile F, Tatsch K, Eschner W (2015). FDG PET/CT: EANM procedure guidelines for tumour imaging: version 2.0. Eur J Nucl Med Mol Imaging.

[17] Nioche C, Orlhac F, Boughdad S, Reuze S, Goya-Outi J, Robert C (2018). LIFEx: a freeware for radiomic feature calculation in multimodality imaging to accelerate advances in the characterization of tumor heterogeneity. Cancer Res.

[18] Kirienko M, Cozzi L, Rossi A, Voulaz E, Antunovic L, Fogliata A (2018). Ability of FDG PET and CT radiomics features to differentiate between primary and metastatic lung lesions. Eur J Nucl Med Mol Imaging.

[19] McNeish DM (2015). Using Lasso for predictor selection and to assuage overfitting: a method long overlooked in behavioral sciences. Multivar Behav Res.

[20] Collins GS, Reitsma JB, Altman DG, Moons KG (2015). BMJ (Clinical research ed).

[21] DeLong ER, DeLong DM, Clarke-Pearson DL (1988). Comparing the areas under two or more correlated receiver operating characteristic curves: a nonparametric approach. Biometrics..

[22] Apostolova I, Ego K, Steffen IG, Buchert R, Wertzel H, Achenbach HJ (2016). The asphericity of the metabolic tumour volume in NSCLC: correlation with histopathology and molecular markers. Eur J Nucl Med Mol Imaging.

[23] Apostolova I, Rogasch J, Buchert R, Wertzel H, Achenbach HJ, Schreiber J (2014). Quantitative assessment of the asphericity of pretherapeutic FDG uptake as an independent predictor of outcome in NSCLC. BMC Cancer.

[24] Hyun SH, Ahn MS, Koh YW, Lee SJ (2019). A machine-learning approach using PET-based radiomics to predict the histological subtypes of lung cancer. Clin Nucl Med.

[25] Oliver JA, Budzevich M, Zhang GG, Dilling TJ, Latifi K, Moros EG (2015). Variability of image features computed from conventional and respiratory-gated PET/CT images of lung cancer. Transl Oncol.

[26] Chae H-D, Park CM, Park SJ, Lee SM, Kim KG, Goo JM (2014). Computerized texture analysis of persistent part-solid ground-glass nodules: differentiation of preinvasive lesions from invasive pulmonary adenocarcinomas. Radiology..

[27] Tsubakimoto M, Yamashiro T, Tamashiro Y, Murayama S (2018). Quantitative CT density histogram values and standardized uptake values of FDG-PET/CT with respiratory gating can distinguish solid adenocarcinomas from squamous cell carcinomas of the lung. Eur J Radiol.

[28] Zhang Y, Qiang JW, Ye JD, Ye XD, Zhang J. High resolution CT in differentiating minimally invasive component in early lung adenocarcinoma, Lung cancer (Amsterdam, Netherlands). 2014;84:236–41. 10.1016/j.lungcan.2014.02.008. 10.1016/j.lungcan.2014.02.008 24679953

[29] Lee HY, Choi YL, Lee KS, Han J, Zo JI, Shim YM (2014). Pure ground-glass opacity neoplastic lung nodules: histopathology, imaging, and management. AJR Am J Roentgenol.

[30] Shao X, Shao X, Niu R, Xing W, Wang Y (2019). A simple prediction model using fluorodeoxyglucose-PET and high-resolution computed tomography for discrimination of invasive adenocarcinomas among solitary pulmonary ground-glass opacity nodules. Nucl Med Commun.

[31] Niu R, Shao X, Shao X, Wang J, Jiang Z, Wang Y (2019). Lung adenocarcinoma manifesting as ground-glass opacity nodules 3 cm or smaller: evaluation with combined high-resolution CT and PET/CT modality. AJR Am J Roentgenol.

[32] Kim TJ, Goo JM, Lee KW, Park CM, Lee HJ (2009). Clinical, pathological and thin-section CT features of persistent multiple ground-glass opacity nodules: comparison with solitary ground-glass opacity nodule. Lung cancer (Amsterdam, Netherlands).

[33] Dai J, Yu G, Yu J (2018). Can CT imaging features of ground-glass opacity predict invasiveness?. A meta-analysis Thoracic cancer.

[34] Yoshizawa A, Sumiyoshi S, Sonobe M, Kobayashi M, Fujimoto M, Kawakami F (2013). Validation of the IASLC/ATS/ERS lung adenocarcinoma classification for prognosis and association with EGFR and KRAS gene mutations: analysis of 440 Japanese patients. J Thorac Oncol.

[35] Han S, Woo S, Suh CH, Kim YJ, Oh JS, Lee JJ. A systematic review of the prognostic value of texture analysis in (18)F-FDG PET in lung cancer. 2018;32:602-610. doi:10.1007/s12149-018-1281-9. 10.1007/s12149-018-1281-9 30014440

