# Supplementary material for: Value of 18F-FDG PET/CT-based radiomics model to distinguish the growth patterns of early invasive lung adenocarcinoma manifesting as ground-glass opacity nodules
Source: EJNMMI Res. 2020 Jul 13;10:80. doi: 10.1186/s13550-020-00668-4 (PMC7359213; doi:10.1186/s13550-020-00668-4)
Supplement: Supplementary file 1 — Supplementary material 1. Report on image processing and radiomic features extraction [file 13550_2020_668_MOESM1_ESM.docx]

**Supplementary material–**Report on image processing and radiomic features extraction

| Acquisition and reconstruction | \| **Acquisition parameters** \| **Siemens Biograph mCT 64** \| \| \| --- \| --- \| --- \| \| **PET** \| **CT** \| \| **^18^F-FDG activity (MBq)*** \| 350-550 (3.70-7.77MBq/kg) \| – \| \| **Min/bed position** \| 2.5 \| – \| \| **Crystal** \| LSO \| – \| \| **Reconstruction** \| OSEM + PSF + TOF \| – \| \| **Matrix (pixels)** \| 200×200 \| 512×512 \| \| **Resolution (mm)** \| 4.0 \| 0.7 \| \| **Slice thickness (mm)** \| 3mm \| 3mm \| \| **Slices** \| – \| 64 \| \| **Voltage (kV)** \| – \| 140 \| \| **Tube current (mA)** \| – \| 64 \| \| **Reconstruction** \| Gaussian filtering with a full-width at half maximum of 2.0mm; 2 iterations and 21 subsets \| B70f very sharp \| \| *Administered activity was calculated according to the European Association of Nuclear Medicine (EANM) guidelines, version 1.0, and from February 2015, version 2.0 [16] \| \| \| |
| --- | --- | --- | --- | --- | --- | --- | --- | --- | --- | --- | --- | --- | --- | --- | --- | --- | --- | --- | --- | --- | --- | --- | --- | --- | --- | --- | --- | --- | --- | --- | --- | --- | --- | --- | --- | --- | --- | --- | --- | --- | --- | --- |
| Approach | The images were analyzed as a volume (3D). |
| Process structure | Image acquisition -> reconstruction ->anonymization -> segmentation -> export -> texture analysis -> feature calculation report |
| Software | LIFEx v5.10  (IMIV, CEA, Inserm, CNRS, Univ. Paris-Sud, Université Paris Saclay, CEA-SHFJ, 91400, Orsay, France) |
| Data availability | All the original patient DICOM files are stored in the institutional PACS. Anonymized DICOM files are stored on the Department's hard disk. The calculations of the features are stored in the repository (link). |

| **Data conversion** | |
| --- | --- |
| Procedure | None |
| **Image post-acquisition processing** | |
| Procedure | None |
| **Segmentation** | |
| ROI | The VOI included the primary tumor lesion. Textural features were calculated on PET and CT images in their respective VOIs. |
| Procedure | The VOIs were semi-automatically defined on PET images with a threshold of 70% or 40% of the maximum standardized uptake value (SUV_max_) using the LIFEx package (version 5.10, http://www.lifexsoft.org). VOIs on CT images were manually delineated and segmented slice-by-slice. Large vessels and bronchus were excluded from the VOI. |
| **Interpolation** |  |
| Voxel dimensions | Not applicable |
| Image interpolation method | Not applicable |
| Image intensity rounding | Not applicable |
| ROI interpolation method | Not applicable |
| ROI partial volume | Not applicable |
| **Re-segmentation** |  |
| ROI mask criteria | None |
| **Discretization** |  |
| Discretisation method | PET: 64 bins from 0 to 20; 64 bins from 0 to 10  CT: 400 bins from -1000 to 3000 |
| **Feature calculation** |  |
| Feature set | \| **Conventional Indices** \| \| \| --- \| --- \| \| **PET** \| **CT** \| \| SUVmin  SUVmean  SUVstd  SUVmax  SUVQ1  SUVQ2  SUVQ3  SUVpeak sphere 0.5mL  SUVpeak sphere 1mL  TLG (mL) \| HUmin  HUmean  HUstd  HUmax  HUQ1  HUQ2  HUQ3 \| \| **First Order Features** \| \| \| SHAPE_Volume (mL)  SHAPE_Volume (#vx)  SHAPE_Sphericity  SHAPE_Compacity  HISTO_Skewness  HISTO_Kurtosis  HISTO_ExcessKurtosis  HISTO_Entropy_log_10_  HISTO_Entropy_log_2_  HISTO_Energy \| \| \| **Second Order Features** \| \| \| **Grey Level Co-occurrence Matrix (GLCM)** \| \| \| GLCM_Homogeneity  GLCM_Energy  GLCM_Contrast  GLCM_Correlation  GLCM_Entropy_log_10_  GLCM_Entropy_log_2_  GLCM_Dissimilarity \| \| \| **Grey-Level Run Length Matrix (GLRLM)** \| \| \| Short-run emphasis (SRE)  Long-run emphasis (LRE)  Low grey-level run emphasis (LGRE)  High grey-level run emphasis (HGRE)  Short-run low grey-level emphasis (SRLGE)  Short-run high grey-level emphasis (SRHGE)  Long-run low grey-level emphasis (LRLGE)  Long-run high grey-level emphasis (LRHGE)  Grey-level non-uniformity for run (GLNU)  Run length non-uniformity (RLNU)  Run percentage (RP) \| \| \| **Neighborhood Grey-Level Different Matrix (NGLDM)** \| \| \| NGLDM_Coarseness  NGLDM_Contrast  NGLDM_Busyness \| \| \| **Grey-Level Zone Length Matrix (GLZLM)** \| \| \| Short-zone emphasis (SZE)  Long-zone emphasis (LZE)  Low grey-level zone emphasis (LGZE)  High grey-level zone emphasis (HGZE)  Short-zone low grey-level emphasis (SZLGE)  Short-zone high grey-level emphasis (SZHGE)  Long-zone low grey-level emphasis (LZLGE)  Long-zone high grey-level emphasis (LZHGE)  Grey-level non-uniformity for zone (GLNU)  Zone length non-uniformity (ZLNU)  Zone percentage (ZP) \| \| |
| Feature parameters | The parameters were calculated according to the formulas described in the software manual (LIFEx v5.10)  (IMIV, CEA, Inserm, CNRS, Univ. Paris-Sud, Université Paris Saclay, CEA-SHFJ, 91400, Orsay, France) |
