# Supplementary material for: Value of 18F-FDG PET/CT-based radiomics model to distinguish the growth patterns of early invasive lung adenocarcinoma manifesting as ground-glass opacity nodules
Source: EJNMMI Res. 2020 Jul 13;10:80. doi: 10.1186/s13550-020-00668-4 (PMC7359213; doi:10.1186/s13550-020-00668-4)

**Table Significance of PET/CT texture features between lepidic group and acinar-papillary group**

|  | Different threshold and higher bound of SUV_max_ | | | |  |  |
| --- | --- | --- | --- | --- | --- | --- |
|  | 70% (0-20) | 70% (0-10) | 40% (0-20) | 40% (0-10) |  | slice-by-slice |
| Features PET | P-value | P-value | P-value | P-value | Features CT | P-value |
| SUVmin | 0.899 | 0.899 | 0.553 | 0.553 | HUmin | 0.130 |
| SUVmean | 0.220 | 0.220 | 0.298 | 0.298 | HUmean | **0.022** |
| SUVstd | **0.083** | **0.083** | **0.065** | **0.065** | HUstd | 0.161 |
| SUVmax | **0.087** | **0.087** | **0.078** | **0.078** | HUmax | 0.996 |
| SUVQ1 | 0.376 | 0.376 | 0.586 | 0.586 | HUQ1 | **0.048** |
| SUVQ2 | 0.331 | 0.331 | 0.371 | 0.371 | HUQ2 | **0.024** |
| SUVQ3 | 0.235 | 0.235 | 0.247 | 0.247 | HUQ3 | **0.025** |
| SUVpeak sphere 0.5mL | 0.361 | 0.361 | 0.247 | 0.247 | —— |  |
| SUVpeak sphere 1mL | 0.822 | 0.822 | 0.791 | 0.791 | —— |  |
| TLG (mL) | 0.255 | 0.255 | 0.193 | 0.193 | —— |  |
| SHAPE_VOLUME (mL) | 0.950 | 0.950 | 0.422 | 0.422 | SHAPE_VOLUME (mL) | 0.515 |
| SHAPE_VOLUME (#vx) | 0.957 | 0.957 | 0.422 | 0.422 | SHAPE_VOLUME (#vx) | 0.496 |
| SHAPE_SPHERICITY | **0.080** | **0.080** | **0.027** | **0.027** | SHAPE_SPHERICITY | 0.808 |
| SHAPE_COMPACITY | 0.785 | 0.785 | 0.815 | 0.815 | SHAPE_COMPACITY | 0.341 |
| HISTO_SKEWNESS | 0.425 | 0.425 | 0.466 | 0.466 | HISTO_SKEWNESS | **0.031** |
| HISTO_KURTOSIS | 0.431 | 0.431 | 0.361 | 0.361 | HISTO_KURTOSIS | **0.029** |
| HISTO_EXCESSKURTOSIS | 0.431 | 0.431 | 0.361 | 0.361 | HISTO_EXCESSKURTOSIS | **0.029** |
| HISTO_ENTROPY_LOG10 | **0.078** | **0.078** | **0.082** | **0.053** | HISTO_ENTROPY_LOG10 | 0.161 |
| HISTO_ENTROPY_LOG2 | **0.078** | **0.078** | **0.082** | **0.053** | HISTO_ENTROPY_LOG2 | 0.161 |
| HISTO_ENERGY | **0.091** | **0.096** | **0.068** | **0.070** | HISTO_ENERGY | **0.094** |
| GLCM_HOMOGENEITY | **0.072** | **0.083** | **0.080** | **0.078** | GLCM_HOMOGENEITY | 0.164 |
| GLCM_ENERGY | **0.082** | **0.085** | **0.065** | **0.059** | GLCM_ENERGY | 0.566 |
| GLCM_CONTRAST | **0.072** | **0.072** | **0.053** | **0.058** | GLCM_CONTRAST | 0.196 |
| GLCM_CORRELATION | 0.712 | 0.683 | 0.228 | 0.243 | GLCM_CORRELATION | 0.800 |
| GLCM_ENTROPY_LOG10 | **0.091** | **0.089** | **0.068** | **0.042** | GLCM_ENTROPY_LOG10 | 0.763 |
| GLCM_ENTROPY_LOG2 | **0.091** | **0.089** | **0.068** | **0.042** | GLCM_ENTROPY_LOG2 | 0.763 |
| GLCM_DISSIMILARITY | **0.074** | **0.070** | **0.061** | **0.074** | GLCM_DISSIMILARITY | 0.147 |
| GLRLM_SRE | **0.069** | **0.070** | 0.102 | **0.094** | GLRLM_SRE | 0.321 |
| GLRLM_LRE | 0.104 | 0.127 | 0.127 | 0.115 | GLRLM_LRE | 0.276 |
| GLRLM_LGRE | 0.420 | 0.431 | 0.408 | 0.448 | GLRLM_LGRE | **0.054** |
| GLRLM_HGRE | 0.176 | 0.176 | 0.210 | 0.206 | GLRLM_HGRE | **0.024** |
| GLRLM_SRLGE | 0.448 | 0.448 | 0.398 | 0.508 | GLRLM_SRLGE | **0.057** |
| GLRLM_SRHGE | 0.156 | 0.183 | 0.180 | 0.203 | GLRLM_SRHGE | **0.024** |
| GLRLM_LRLGE | 0.307 | 0.371 | 0.336 | 0.392 | GLRLM_LRLGE | **0.054** |
| GLRLM_LRHGE | 0.420 | 0.206 | 0.387 | 0.317 | GLRLM_LRHGE | **0.025** |
| GLRLM_GLNU | 0.117 | 0.122 | 0.361 | 0.298 | GLRLM_GLNU | 0.167 |
| GLRLM_RLNU | 0.193 | 0.408 | 0.109 | 0.186 | GLRLM_RLNU | 0.508 |
| GLRLM_RP | **0.082** | **0.089** | 0.115 | **0.098** | GLRLM_RP | 0.307 |
| NGLDM_COARSENESS | 0.336 | 0.336 | 0.134 | 0.213 | NGLDM_COARSENESS | 0.478 |
| NGLDM_CONTRAST | 0.115 | **0.070** | **0.093** | 0.145 | NGLDM_CONTRAST | 0.028 |
| NGLDM_BUSYNESS | **0.059** | 0.259 | 0.280 | 0.193 | NGLDM_BUSYNESS | 0.220 |
| GLZLM_SZE | **0.055** | **0.061** | **0.077** | **0.031** | GLZLM_SZE | 0.371 |
| GLZLM_LZE | **0.047** | **0.085** | 0.147 | 0.102 | GLZLM_LZE | 0.196 |
| GLZLM_LGZE | 0.431 | 0.219 | **0.084** | 0.272 | GLZLM_LGZE | **0.046** |
| GLZLM_HGZE | **0.090** | 0.128 | **0.072** | 0.120 | GLZLM_HGZE | **0.026** |
| GLZLM_SZLGE | 0.815 | 0.164 | 0.838 | 0.808 | GLZLM_SZLGE | **0.047** |
| GLZLM_SZHGE | **0.037** | **0.061** | **0.046** | **0.061** | GLZLM_SZHGE | **0.026** |
| GLZLM_LZLGE | 0.137 | 0.139 | 0.164 | 0.124 | GLZLM_LZLGE | **0.041** |
| GLZLM_LZHGE | **0.070** | 0.213 | 0.167 | 0.134 | GLZLM_LZHGE | **0.052** |
| GLZLM_GLNU | 0.271 | 0.291 | 0.576 | 0.623 | GLZLM_GLNU | 0.196 |
| GLZLM_ZLNU | **0.012** | **0.065** | **0.024** | **0.030** | GLZLM_ZLNU | 0.690 |
| GLZLM_ZP | **0.044** | **0.083** | 0.111 | **0.078** | GLZLM_ZP | 0.251 |

Bold text indicates P <0.1.

**Different SUV threshold PET-score calculation formula and ROC curve**

PET-score-70% = − 2.11038 × SHAPE_Sphericity + 0.0035 × GLZLM_SZHGE − 0.00005 × GLZLM_LZHGE + 0.14261 × GLZLM_ZLNU


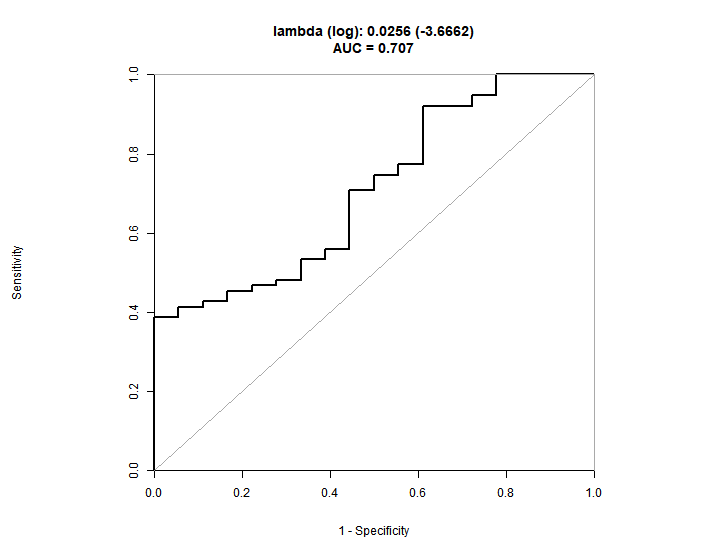


PET-score-40% = − 6.10801 × SHAPE_Sphericity + 0.16396 × GLZLM_ZLNU


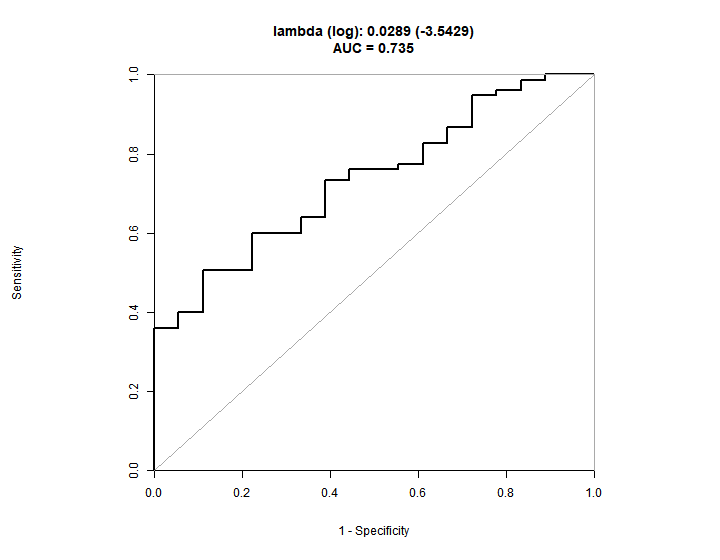


**Different SUV higher bound PET-score calculation formula and ROC curve**

PET-score-20 = − 6.10801 × SHAPE_Sphericity + 0.16396 × GLZLM_ZLNU


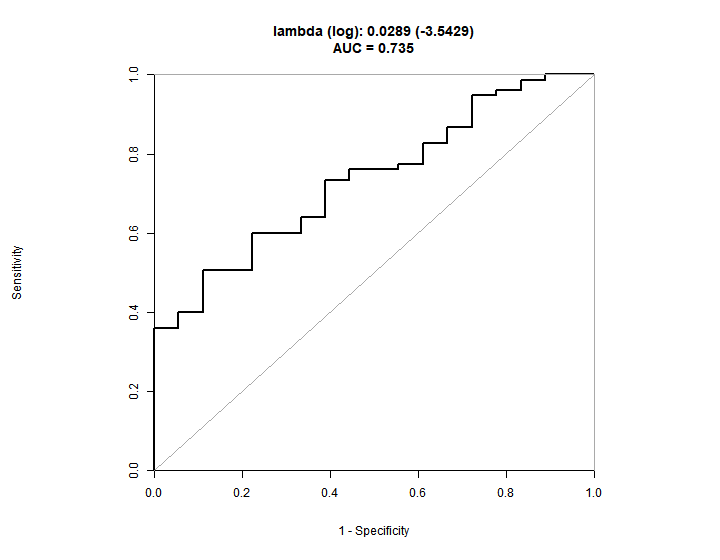


PET-score-10 = − 5.6526 × SHAPE_Sphericity + 1.27934 × GLZLM_SZE + 0.0028 × GLZLM_SZHGE


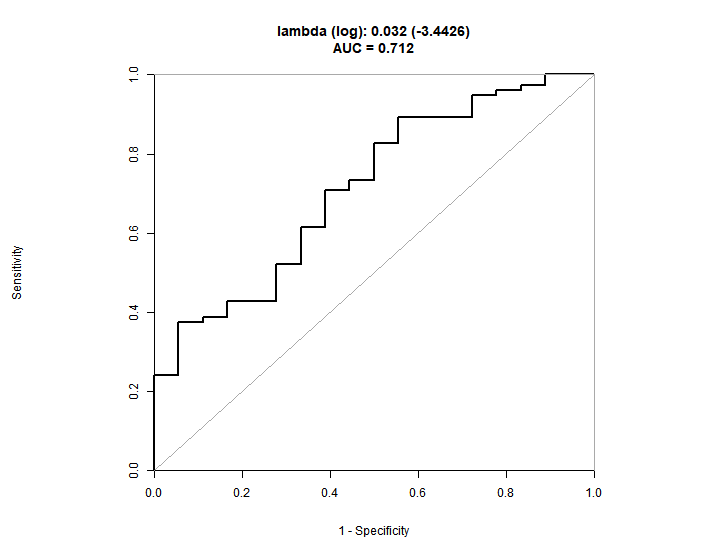

Supplement: Supplementary file 2 — Supplementary material 2. Significance of PET/CT texture features between lepidic group and acinar-papillary group [file 13550_2020_668_MOESM2_ESM.docx]
